# Dynamic change in Siglec-15 expression in peritumoral macrophages confers an immunosuppressive microenvironment and poor outcome in glioma

**DOI:** 10.3389/fimmu.2023.1159085

**Published:** 2023-05-10

**Authors:** Quan Chen, Bingkun Chen, Chunhua Wang, Li Hu, Qiongwen Wu, Yanyang Zhu, Qiuyu Zhang

**Affiliations:** ^1^Institute of Immunotherapy, Fujian Medical University, Fuzhou, China; ^2^Department of Oncology, Fujian Medical University Union Hospital, Fuzhou, China; ^3^Department of Immunology, The School of Basic Medical Sciences, Fujian Medical University, Fuzhou, China

**Keywords:** Siglec-15, glioma, macrophage, combination therapy, tumor microenvironment, PD-L1

## Abstract

**Background:**

Sialic acid-binding immunoglobulin-like lectin-15 (Siglec-15) was reported to be a novel immune checkpoint molecule comparable to programmed cell death 1 ligand 1 (PD-L1). However, its expression profile and immunosuppressive mechanisms in the glioma tumor microenvironment have not yet been fully explored.

**Objectives:**

To identify the expression profile and potential function of Siglec-15 in glioma tumor microenvironment.

**Methods:**

We investigated Siglec-15 and PD-L1 expression in tumor tissues from 60 human glioma patients and GL261 tumor models. Next, Siglec-15 knockout macrophages and mice were used to elucidate the immunosuppressive mechanism of Siglec-15 impacting macrophage function.

**Results:**

Our results demonstrated that high levels of Siglec-15 in tumor tissues was positively correlated with poor survival in glioma patients. Siglec-15 was predominantly expressed on peritumoral CD68^+^ tumor-associated macrophages, which accumulated to the highest level in grade II glioma and then declined as grade increased. The Siglec-15 expression pattern was mutually exclusive with that of PD-L1 in glioma tissues, and the number of Siglec-15^+^PD-L1^-^ samples (n = 45) was greater than the number of Siglec-15^-^PD-L1^+^ samples (n = 4). The dynamic change in and tissue localization of Siglec-15 expression were confirmed in GL261 tumor models. Importantly, after *Siglec15* gene knockout, macrophages exhibited enhanced capacities for phagocytosis, antigen cross-presentation and initiation of antigen-specific CD8^+^ T-lymphocyte responses.

**Conclusion:**

Our findings suggested that Siglec-15 could be a valuable prognostic factor and potential target for glioma patients. In addition, our data first identified dynamic changes in Siglec-15 expression and distribution in human glioma tissues, indicating that the timing of Siglec-15 blockade is critical to achieve an effective combination with other immune checkpoint inhibitors in clinical practice.

## Introduction

Gliomas are the most common and aggressive primary brain tumors associated with a very poor prognosis, especially glioblastoma (GBM), with a 5-year overall survival rate below 5% (1). Conventional therapeutic strategies for GBM are unsatisfactory. Immune checkpoint inhibitors (ICIs) targeting PD-1/PD-L1 alone have produced a demonstrable therapeutic benefit in several tumor types; however, their efficacy in gliomas has not been satisfactory (2, 3). Accumulating data suggest that the heterogeneous tumor microenvironment (TME) is one of the important determinants of immunotherapeutic efficacy (4, 5). The importance of tumor-associated macrophages (TAMs) to glioma progression is highlighted by their large numbers within tumor sites, with TAMs comprising up to 50% of the cells in the tumor mass (6, 7). TAMs are a heterogeneous population encompassing M1-polarized macrophages with proinflammatory properties and more M2-like macrophages that may promote tumor growth (8, 9). Notably, predominantly M2-polarized TAMs are found in primary late-stage human tumors and relapse samples from glioma patients (7). In fact, due to resistance to therapies targeting PD-1/PD-L1, controlling the immunosuppressive activity of TAMs has emerged as a novel promising strategy for immunotherapy, such as targeting colony stimulating factor 1 receptor (CSF1R) and CD47 (10–12). However, the mechanism underlying macrophage polarization and immunosuppressive activity in brain tumors has not been completely elucidated.

Recently, Siglec-15, a member of the sialic acid-binding immunoglobulin-type lectins (Siglecs), has emerged as an immune suppressor and a potential target for immunotherapy (13–15). Unlike most other Siglec family members, Siglec-15 is expressed primarily by macrophages and dendritic cells (13, 16). Previous studies have reported that Siglec-15 expression is mutually exclusive to B7-H1 (PD-L1) in TME of several cancer types, including non-small cell lung cancer (NSCLC), head and neck squamous cell carcinoma (HNSCC), breast and bladder cancers (13, 17), indicating that Siglec-15 works independently from the PD-L1/PD-1 pathway to restrain antitumor immune responses. However, whether Siglec-15 expression is involved in M2 polarization and confers resistance to anti-PD-1/PD-L1 therapy in glioma has yet to be elucidated. Here, we analyzed the correlation between the Siglec-15 expression levels and survival outcomes of glioma patients based on The Cancer Genome Atlas (TCGA) database. Next, we detected the dynamic changes in Siglec-15 expression in TME at different grades of human glioma and in the GL261 mouse model. We further investigated the potential role of Siglec-15 in macrophage polarization and the initiation of a T-cell response, using *Siglec15* gene knockout mice and macrophages. Our findings revealed that Siglec-15 might be a prognostic factor and immune target for patients with glioma.

## Results

### Siglec-15 expression associated with tumor-infiltrating macrophages contributes to worse clinical outcome in human glioma

**Figure 1:**
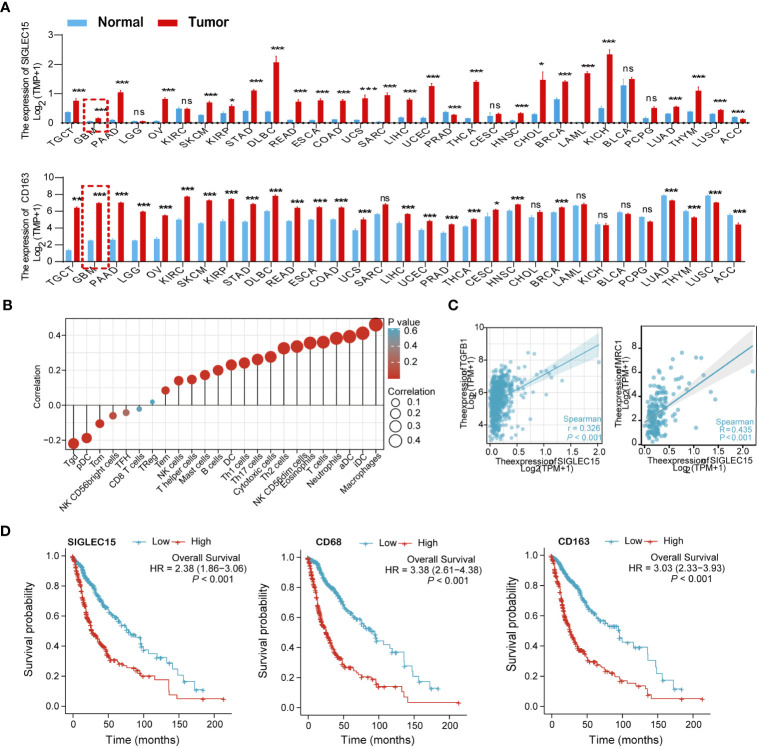
Relationships of SIGLEC15 expression with the infiltration of tumor-infiltrating macrophages were assessed in human glioma based on the TCGA and GTEx databases. **(A)** The mRNA expression levels of CD68 (upper graph) and SIGLEC15 (lower graph) in 31 types of human cancers and corresponding normal tissues were assessed by meta-analysis. **(B)** Correlations of SIGLEC15 mRNA expression with the infiltration levels of different immune cell types are shown in a bubble chart. Bubble size represents the correlation coefficient value, and bubble color represents the P value. **(C)** The correlation of mRNA expression levels between SIGLEC15 and genes encoding M2-like macrophage markers (MRC1 and TGFB1) were assessed by meta-analysis of 698 glioma patients. **(D)** Associations of mRNA expression levels with survival in glioma patients. The expression levels of SIGLEC15, CD68 and CD163 were binned into high and low based on the median cutoff value (n = 695), and survival curves were compared using the log-rank (Mantel–Cox) test. ns, no significance; *P< 0.05; ***P< 0.01; ***P< 0.001.

To assess the association of Siglec-15 expression with tumor-infiltrating macrophages, we analyzed the gene expression levels of Siglec-15 and tumor-infiltrating macrophage markers (CD68 and CD163) in 31 types of tumor tissues from the TCGA and GTEx databases. The data showed that the mRNA expression levels of CD68, CD163 and SIGLEC15 were higher in tumor tissues than in normal tissues (**Figure 1A**; **Supplementary Figure 1A**) across a broad spectrum of human cancers, including glioma. Notably, based on the 24 well-established immune-associated gene sets, macrophages showed the highest correlation with SIGLEC15 expression (**Figure 1B**). Consistent with this result, SIGLEC15 expression in 698 glioma tissues samples was positively correlated with the gene expression of M2-like macrophage markers, including C-type mannose receptor 1 (MRC1, also known as CD206) and Transforming growth factor 1 (TGFB1) (**Figure 1C**). Next, 695 glioma patients were divided into two groups based on median expression levels of SIGLEC15 (high and low SIGLEC15 expression groups). The data showed that SIGLEC15 expression level was negatively associated with the survival rates of glioma patients, including those for overall survival (OS), disease-free survival (DFS) and progression-free interval (PFI) (**Figure 1D**; **Supplementary Figures 1B, C**).

### Siglec-15 expression in peritumoral macrophages changed with tumor development and progression in human glioma and murine GL261 allografts

**Figure 2:**
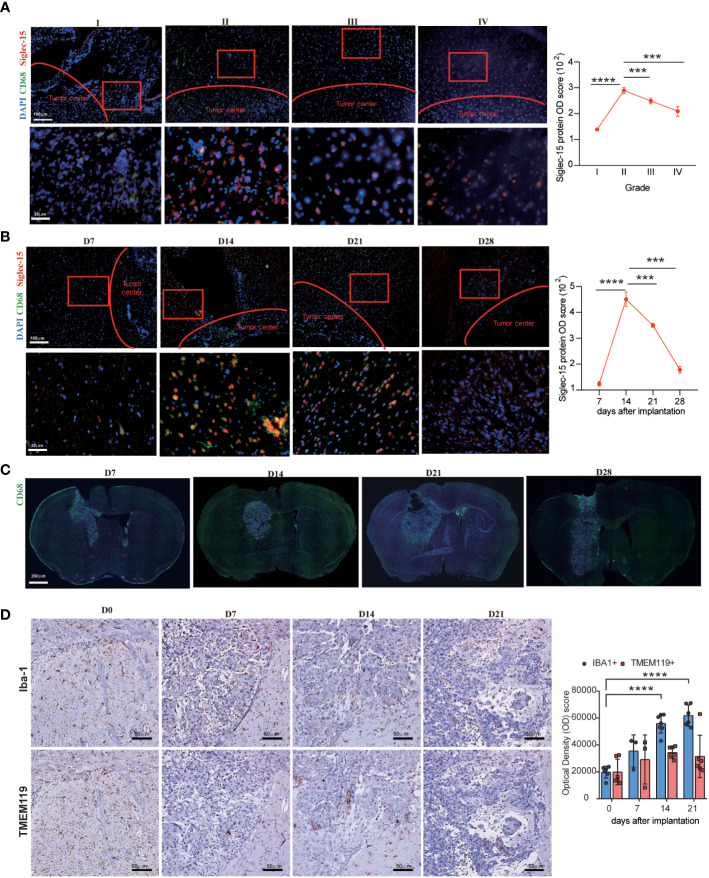
Dynamic change in Siglec-15 expression in TAMs in human glioma and GL261 murine glioma assessed by immunofluorescence staining. Left graph: **(A)** Representative images and Optical density (OD) score of Siglec-15 expression in human glioma tissues from grade I to grade IV. **(B–D)** Representative images and OD score of Siglec-15, CD68, TMEM119 and Iba1 in allografts tissue on the indicated days after tumor implantation. C57BL/6 mice were intracranially injected with GL261 cells, tumor tissues were collected on (D7, D14, D21, D28) to staining target protein. Siglec-15 and CD68 were stained by immunofluorescence staining **(B, C)**, and TMEM119 and Iba1 were stained by IHC **(D)**. Right graph: OD scores of target gene expression were quantified by ImageJ software. Scale bar: 250 μm, original magnification 40×; 100 μm, original magnification 100×; 50 μm, original magnification 200×; 25 μm, original magnification 400×. Data are representative of three independent experiments. *** *P*< 0.001; **** *P*< 0.0001.

Considering that the different expression patterns of immune regulators confer their different functions, the location of Siglec-15 expression in tumor tissues was examined by multiple-color immunofluorescence staining. The results indicated that Siglec-15 was expressed in the periphery of tumor cell conglomerates in both human glioma tissues and murine GL261 allografts. In addition, Siglec-15 was predominantly expressed on CD68-positive TAMs (**Figure 2A**). Importantly, Siglec-15 expression on TAMs was dynamic during tumor progression. Siglec-15 expression were detected in grade I tissues, and the levels peaked in grade II tissues and then declined rapidly to low levels in grade III and IV tissues. Similar dynamic change in Siglec-15 and CD68 levels were found in GL261 allografts, with the peak expression level observed on Day 14 after tumor implantation (**Figures 2B, C**). Furthermore, we examined the dynamic change in TMEM119^+^ microglia and Iba1^+^ macrophages in the brain of GL261-bearing mice. Different from Iba1^+^ macrophage, TMEM119^+^ microglia was not accumulated during tumor progression, indicating that Siglec-15 was predominantly expressed in peripheral monocyte-derived macrophages of glioma TME (**Figure 2D**). To substantiate the relationship between Siglec-15 expression and T-cell infiltration, triple immunofluorescence staining for CD3, CD68 and Siglec-15 was performed in tissue samples of human glioma and murine GL261. The negative correlation between CD3 and Siglec-15 expression was found in both human glioma and mouse GL261 model samples (**Supplementary Figures 2A, B**). The infiltration of CD4 and CD8 T cells in GL261 allografts was lowest when Siglect15 reached the peak expression level on Day 14 (**Supplementary Figure 2C**).

### Siglec-15 and PD-L1 expression was mutually exclusive in the glioma TME

**Figure 3:**
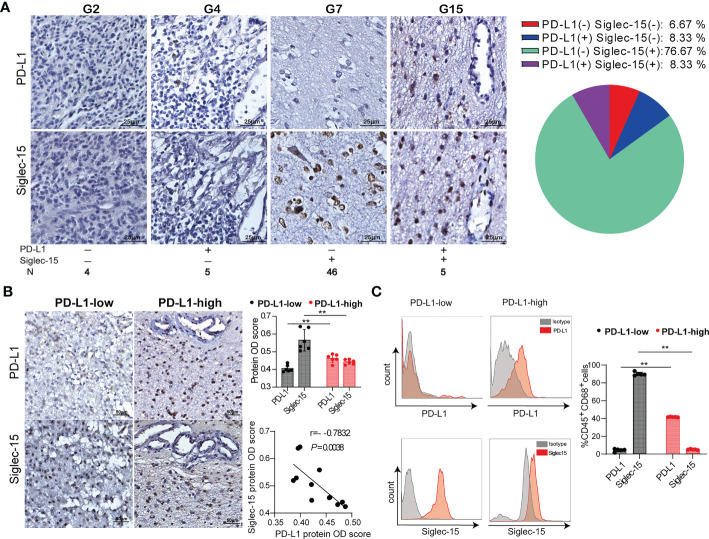
The negative correlation of Siglec-15 expression with PD-L1 expression in human glioma tissues. **(A)** Representative images of immunohistochemical staining for PD-L1 and Siglec-15 in human glioma tissues (left graph). Scale bar = 25 μm. The percentages of the four phenotype defined populations based on Siglec-15 and PD-L1expression profiles in tumor tissues were analyzed in 60 glioma patients (right graph). **(B)** The correlation of PD-L1 and Siglec-15 expression in glioma tissue. PD-L1 expression levels were classified into low and high groups by the median OD score. Scale bar = 50 μm. **(C)** PD-L1 and Siglec-15 expression on CD45^+^CD68^+^ TAMs in human glioma was analyzed by flow cytometry. PD-L1 expression levels were divided into low and high groups by the median MFI (mean fluorescence intensity). G2, G4, G5 and G7: four representative tumor tissue samples. ** *P*< 0.01.

We specifically investigated the immunohistochemical staining patterns of Siglec-15 and PD-L1. A total of 46/60 cases (76.67%) showed a pattern with single positive staining for Siglec-15, and it was significantly more frequent than single positive staining for PD-L1 (5/60, 8.33%), double positive staining (5/60, 8.33%) or double negative staining (4/60, 6.67%) (**Figure 3A**). According to the IRS-classification scoring systems, high and low expression of Siglec-15 was defined by the cutoff value of the median protein OD score. Notably, Siglec-15 expression was significantly lower in tumor tissues with high levels of PD-L1 expression than in those with low PD-L1 expression. There was a significant inverse correlation between the expression of Siglec-15 and PD-L1 in tumor tissues (**Figure 3B**). Similar results were obtained with tumor tissues from the mouse GL261 model (**Supplementary Figure 3A**). The inverse correlation between PD-L1 and Siglec-15 was also confirmed in fresh human glioma tissues, for which the cell-surface expression levels of PD-L1 and Siglec-15 on TAMs were analyzed by flow cytometry (**Figure 3C**). The results of glioma database analysis also showed that the SIGLEC15 expression level was negatively associated with that of PD-L1 in patients with GBM or low-grade glioma (LGG) (**Supplementary Figure 3B**). Overall, Siglec-15 expression was mutually exclusive with PD-L1 expression in the glioma TME.

### Siglec-15 expression in macrophages was upregulated by soluble cytokines released from tumor cells but not T cells

**Figure 4:**
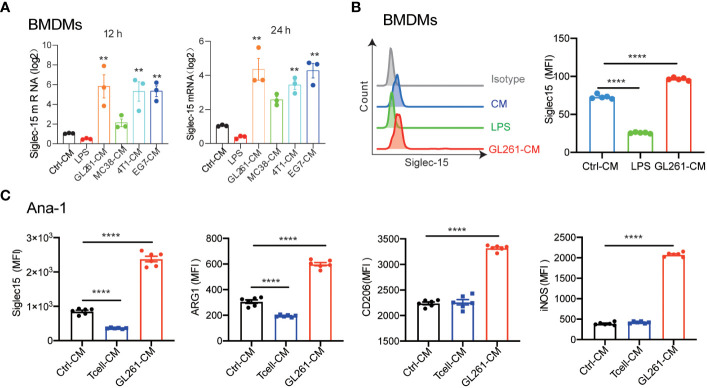
Induction of Siglec-15 expression on Macrophages by soluble proteins released from tumor cells. **(A)** Siglec-15 expression in BMDMs was induced by CM from various types of tumor cell lines. BMDMs were incubated with 1μg/ml LPS, control medium or conditioned medium from 4T1, GL261, MC38 or EG-7 cells for 12 h and 24 h, and *Siglec15* mRNA levels were determined by RT qPCR. **(B)** Siglec-15 expression on BMDMs in the presence of LPS or cell medium of GL261 was analyzed by flow cytometry. **(C)** The expression of Siglec-15, ARG1, CD206 and iNOS on Ana-1 cells was detected by flow cytometry. Ana-1 cells were cultured in the presence of cell medium from GL261 or activated T cells (pretreated with 1 μg /ml anti-mouse CD3) for 24 h. CM: cell culture medium, MFI: mean fluorescence intensity. The data represent the mean ± SEM and are from two to three independent experiments. ** *P*< 0.01; **** *P*< 0.0001.

To further elucidate whether Siglec-15 expression is regulated by tumor cells or T cells, we detected macrophage Siglec-15 expression levels in the presence of cell culture medium (CM) from tumor cells or T cells. As expected, Siglec-15 expression was upregulated in both bone marrow derived macrophages (BMDMs) and peritoneal macrophages (PEMs) after treatment with CM from 4T1, GL261, MC38 or EG7 cells. However, its expression was downregulated after treatment with lipopolysaccharides (LPS) or CM from T cells (**Figures 4A, B**; **Supplementary Figures 4A, B**). To further examine whether Siglec-15 expression influences macrophage phenotype and function, we evaluated the expression of arginase-1 ARG1, MRC1/CD206 and inducible nitric oxide synthase (iNOS) in Ana-1 cells, a murine macrophage cell line that constitutively expresses Siglec-15. Compared with control treatment, CM of GL261 but not CM of T cells upregulated the expression of Siglec-15, ARG1, CD206, and iNOS (**Figure 4C**). Together, our results support soluble factors released by tumor cells but not T cells regulate Siglec-15 expression in macrophages.

### Siglec-15 directed the differentiation and polarization of macrophages mediating suppression of the T-cell response

**Figure 5:**
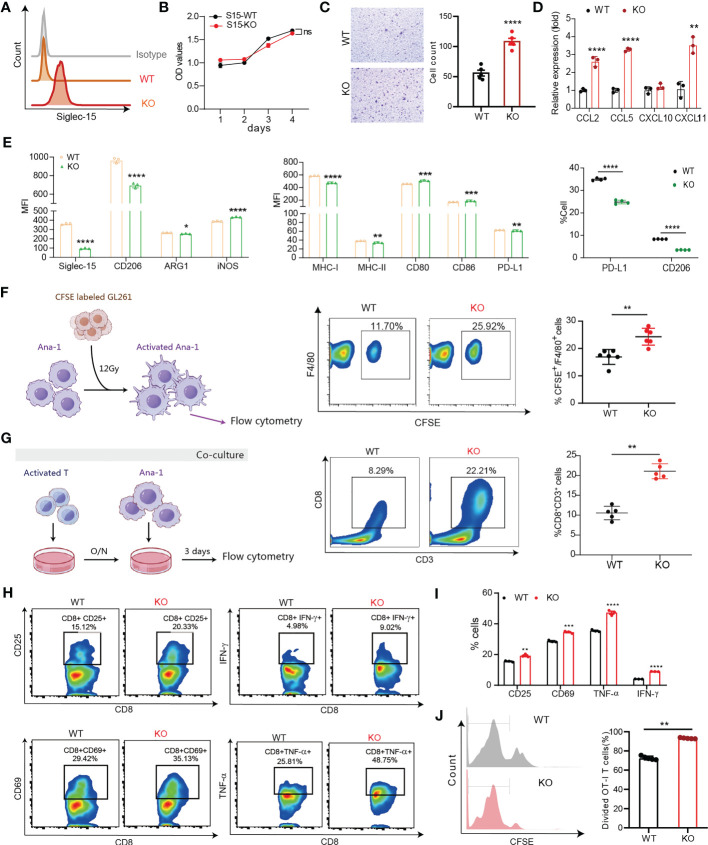
Siglec-15 expression on macrophages inhibited their migration, M1-like polarization, phagocytosis and T-cell priming activity. **(A)** Siglec-15-knockout and control mouse macrophage cell lines (Ana-1-KO and Ana-1-WT) were generated and confirmed by flow cytometry (left graph). **(B)** The proliferative capability of Ana-1-KO and Ana-1-WT cells was analyzed by CCK-8 assay. **(C)** Transwell assay was used to detect the migration ability of Ana-1-KO and Ana-1-WT cells. Representative images and quantified data were shown in the left and right graphs, respectively. Scale bar = 100 μm. **(D)** The mRNA expression levels of CC and CXC chemokine in two cell lines were determined by RT qPCR. **(E)** The expression levels of M2-like polarization macrophage markers and antigen presenting associated molecules in two cell lines were compared (left and middle graphs). The CD206 and PD-L1 expression levels on two cell lines were detected by flow cytometry after treatment with GL261 culture media (right graph). **(F)** The phagocytic activity of Ana-1-KO and Ana-1-WT (F4/80^+^) cells was analyzed after coculture with CFSE-labeled GL261 cells pretreated with 8 Gy irradiation for 24 h. **(G)** The frequency of CD8^+^CD3^+^ T cells was analyzed after stimulation with 0.1 μg/ml of anti-CD3 plus anti-CD28 antibodies following coculture with Ana-1-KO or Ana-1-WT cells for 72 h. **(H–J)** The percentage of divided OT-I T cells was counted after coculture with OVA_257-264_-preloaded Ana-1-KO or Ana-1-WT cells for 3 days **(J)**, and the percentages of activated T cells (gating CD3^+^CD8^+^ T cells) were analyzed by flow cytometry in two groups, including CD8^+^CD25^+^, CD8^+^CD69^+^, CD8^+^TNF-α^+^ and CD8^+^IFN-γ^+^ T cells **(H, I)**. MFI: mean fluorescence intensity. The data represent the mean ± SD. * *P*<0.05; ** *P*< 0.01; *** *P*< 0.001; ^###^ *P*< 0.001; **** *P*< 0.0001.

Previous data supported that Siglec-15 might influence the tumor-infiltrating T-cell population (13). Next, we generated *Siglec15* gene knockout Ana-1 cell lines (Ana-1-KO) to investigate the role of Siglec-15 on the function of tumor infiltrating macrophages (**Figure 5A**). No difference in the proliferative capability was found between Ana-1-KO cells and Ana-1-WT cells (**Figure 5B**). Notably, migratory responsiveness to serum was markedly increased after *Siglec15* gene knockout (**Figure 5C**), and Ana-1-KO cells expressed high mRNA levels of chemokine ligands CCL2, CCL5 and CXCL11 in comparison with Ana-1-WT cells (**Figure 5D**). In addition, upregulation of ARG1, iNOS and CD206 expression induced by CM from GL261 cells was impaired in Ana-1-KO cells, suggesting a role for Siglec-15 in regulating the polarization of tumor-associated macrophages (**Figure 5E**). Ana-1-KO cells showed a greater phagocytic ability than Ana-1-WT cells after coculture with irradiated CFSE-labeled GL261 cells (**Figure 5F**). Moreover, the ability of mouse T cells to undergo cell division in response to CD3-CD28 signaling was increased in the presence of Ana-1-KO cells than Ana-1-WT cells (**Figure 5G**). Similarly, coculture with Ana-1-KO cells incubated with the OVA257-264 peptide enhanced the proliferation, activation and cytokine release (TNF-α and IFN-γ) of OT-I T cells compared with their coculture of Ana-1-WT counterparts (**Figures 5H–J**). Furthermore, RNA-seq analysis revealed that the expression of antigen-processing-and-presentation related genes was upregulated in Ana-1-KO cells (**Supplementary Figure 5**). Altogether, our results suggested that the expression of Siglec-15 restricted to macrophages suppressed T-cell response by impairing a series of events, including phagocytosis of apoptotic tumor cells, antigen presentation to T cell, and M2-like polarization in the TME of glioma.

### Siglec-15 expression in peritumoral macrophages conferred primary resistance to anti-PD-1 therapy and radiotherapy

**Figure 6:**
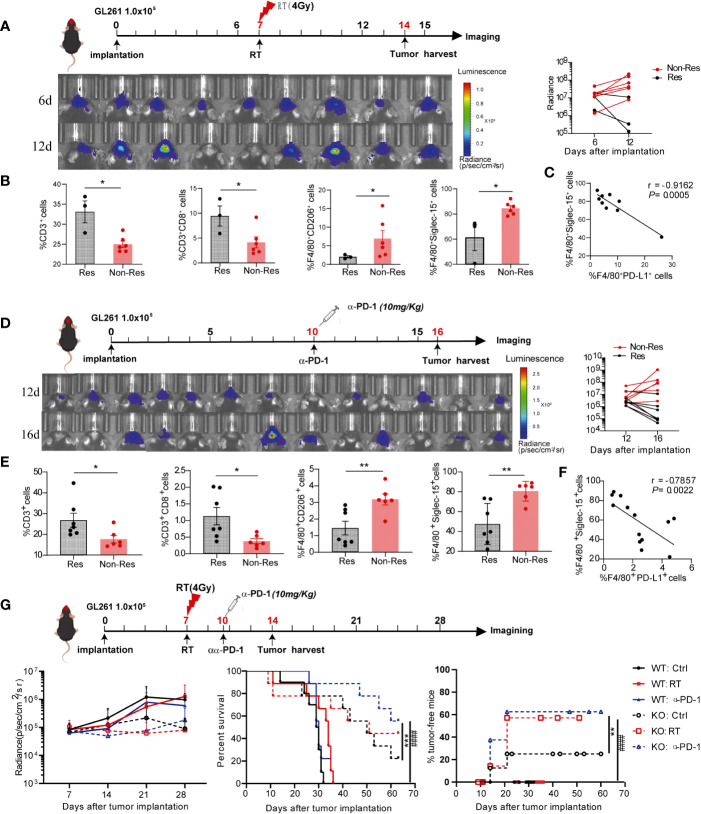
High Siglec-15 expression levels of peritumoral macrophages in GL261 tissue resistant to treatment with anti-PD-1 therapy and radiation therapy (RT). C57BL/6 mice were intracranially injected with firefly luciferase-transgenic GL261 cells, and then tumors were subjected 4 Gy irradiation or mice received an anti-PD-1 antibody (200μg/mouse) on the indicated day. *In vivo* bioluminescent imaging and computer-obtained luminescence (photons/second) data were used to monitor tumor growth and examine treatment response. **(A, D)** Scheme for treatment (upper graph), tumor imaging (lower left graph) and the growth curve of an individual mouse after tumor inoculation (lower right graph). **(B, E)** Tumor-infiltrating immune cells were analyzed in response group and nonresponse group by flow cytometry 8 days after 4Gy radiation therapy **(B)** or 10 days after the first dose of anti-PD-1 therapy **(E)**. **(C, F)** Correlation between the percentages of Siglec-15- and PD-L1-expressing cells in F4/80^+^ macrophages. **(G)** *Siglec-15*-knockout mice and their wild-type littermates were implanted with GL261 cells, and subsequently treated with radiation therapy on Day 6 or anti-PD-1 on Day 10. The tumor growth curve of an individual mouse and the survival rates of mice in six groups are shown (n = 7). * *P*<0.05; ** *P*< 0.01; *** *P*< 0.001; ^###^ *P*< 0.001.

Given that the infiltration of tumor-associated macrophage is essential for anticancer therapy resistance, we further evaluated whether macrophages-derived Siglec-15 imparts resistance to radiotherapy and anti-PD-1 therapy in glioma. After treatment with ionizing irradiation (RT) or anti-PD-1 antibodies, GL261-bearing mice were divided into a non-response group (Non-res) with increased tumor volume and response group (Res) with reduced tumor volume (**Figures 6A, D**). Next, tumor-infiltrating immune cells and Siglec-15 expression were analyzed between the two groups. As shown in **Figures 6B**, **E**, a significantly increased percentage of CD3^+^CD8^+^T cells was found in the Res groups compared to the Non-res group; meanwhile, the percentage of F4/80^+^CD206^+^ and F4/80^+^Siglec-15^+^ macrophages were lower in Res than Non-res. There was a negative correlation between percentage of F4/80^+^PD-L1^+^ and F4/80^+^Siglec-15^+^ macrophages in TME of all mice (**Figures 6C, F**). These results indicated that Siglec-15 might act as a novel TAM-related immune-checkpoint, which is independent of PD-L1/PD-1 pathway. Based on these findings, we further evaluated the therapeutic response of anti-PD-1 therapy or irradiation in GL261-bearing S15^KO^ and S15^WT^ mice. Although no significant difference in tumor growth was found among all groups, both anti-PD-1 therapy and irradiation greatly extended tumor-free survival in S15^KO^ mice compared with S15^WT^ mice. Additionally, anti-PD-1-treated S15^KO^ mice (KO: α-PD-1) showed a higher percentage of survival rate and greater tumor-free percentage than S15^KO^ mice without treatment (KO: Ctrl). (**Figure 6G**). These data strongly suggested that removing the influence of Siglec-15 could greatly improve the outcome of anti-PD-1 therapy in combination with radiotherapy in murine glioma model. Altogether, our results indicate that Siglec-15 expression in peritumoral macrophages may confer primary resistance to antitumor therapy.

## Discussion

In recent years, immune checkpoint inhibitors that target PD-1/PD-L1 and/or cytotoxic T lymphocyte-associated antigen-4 (CTLA-4) have emerged as effective immunotherapeutic agents in several types of cancer, profoundly changing the prognosis of a fraction of patients. The limited number of responders have encouraged researchers to explore novel approaches to overcome resistance to targeting the PD-1/PD-L1 axis. Currently, Siglecs and their interacting sialoglycans have been described as a novel immune checkpoint axis that promotes cancer immune evasion (13, 18–20). Siglecs are type 1 transmembrane proteins displaying an amino-terminal V-like Ig domain and varying numbers of C2-set immunoglobulin domains. They can be divided into two groups based on sequence similarity and evolutionary conservation, and these groups are the conserved Siglecs (Siglec-1, -2, -4, and-15) and the CD33-related Siglecs (Siglec-3, -5, -6, -7, -8, -9, -10, -11, -14, and-16) (21, 22). Previous studies have demonstrated upregulation of Siglec-1, -9, -10, and -14 expression in a high-risk group of patients with glioma and the positive correlation of these four Siglecs with anti-inflammatory cytokine levels in the glioma TME (20). Siglec-3, Siglec -5, Siglec -7 and Siglec-9 were found to be expressed mainly on myeloid-derived suppressor cells (MDSCs) in the glioma TME, and Siglec-3 promotes the expansion and suppression of MDSCs (23). Single-cell sequencing analysis revealed that the expression profile of Siglec-5, Siglec-7, and Siglec-9 were mutually exclusive with that of Siglec-16 in the glioma TME, and that Siglec-16 might suppress antitumor immunity *via* mechanisms different from PD-1, PD-L1 and CTLA4 (24). Similar to that of Siglec-16, the Siglec-15 expression profile was reported to be mutually exclusive to PD-L1 in tumor tissues of non-small cell lung cancer (NSCLC) (13). Given that Siglec-15 has a function complementary to that of PD-1/PD-L1, therapeutic antibody targeting Siglec-15 (NC318) has been tested in a phase I trial for NSCLC patients refractory to PD-1 blockade (25). Although Siglec-15 has been identified as a potential target for cancer immunotherapy, its expression profile in glioma and its correlation with glioma prognosis have not been completely elucidated.

Studies from several research groups have demonstrated that high Siglec-15 expression is found in the TME and that Siglec-15 is predominantly expressed on TAMs. However, the prognostic value of Siglec-15 is heterogeneous among different types of cancers (26–29). Some studies have suggested that Siglec-15 expression on peritumoral macrophages corresponds with a favorable outcome in patients with primary central nervous system lymphoma (PCNSL) or pancreatic ductal adenocarcinoma (PDAC) (16, 26). Nevertheless, previous work by others have suggested that Siglec-15 expression is not associated with the clinicopathologic characteristics or outcome in resectable NSCLC and poorly differentiated invasive ductal breast cancers (17, 29). In this study, we performed a meta-analysis of Siglec-15 and TAM marker expression patterns in 31 types of cancers using RNA-seq data from the TCGA and GTEx. The data showed that SIGLEC15, CD68 and CD163 gene expression was broadly upregulated in tumor tissues compared with normal tissues. High levels of SIGEC15 gene and protein expression were detected in both LGG and GBM tumor tissues, and median survival was shorter for patients with high Siglec-15 expression than patients with low Siglec-15 expression. Importantly, the Siglec-15 expression level was positively correlated with macrophage infiltration in glioma and phenotypic markers of M2-like macrophages, including CD206 and TGFB1. These findings indicated that Siglec-15 could serve as a poor prognostic biomarker involved in the regulation of TAM function in glioma progression.

Based on the unexpected association of Siglec-15 expression with clinical outcome in glioma, we focused our investigation on the dynamic change in Siglec-15 expression in glioma TME identified by multiplexed immunohistochemistry. We found that Siglec-15 was coexpressed with CD68 in the TAMs localized in peritumoral tissues, and that TAMs accumulated to the highest frequency in grade II glioma but then gradually declined in grade III and IV. We validated this finding using GL261 tumor tissue samples collected at different time points after tumor implantation, confirming the dynamic change in the density of Siglec-15-positive TAMs during glioma progression with a high population in the early stage. These findings indicated that identifying the optimal intervention time is important for immunotherapeutic targeting Siglec-15 to achieve the desired efficacy. Similar to many other B7 family members, Siglec-15 shows high sequence and structural homology with PD-L1. Consistent with the results in NSCLC (13), the expression of Siglec-15 and PD-L1 was mutually exclusive in human glioma tissues. An inverse correlation between PD-L1 and Siglec-15 expression was found in both GBM and LGG tissues by RNA-seq and confirmed by IHC staining and flow cytometry. Notably, the expression pattern PD-L1 (-)Siglec-15(+) accounted for the vast majority (76.67%) of 60 glioma patients, and PD-L1(+) expression pattern was detected in only 16.67% of glioma tissues. These results suggested that Siglec-15 mediated immunosuppression might be one of the dominant immune escape mechanisms in the glioma TME.

Increasing evidence shows that tumor-infiltrating macrophages account for up to one-third of late-stage tumor masses and actively support tumor growth, invasion, and angiogenesis (7, 30–32). Our previous study also showed that CD68^+^ TAMs constitute a major component of the tumor stroma with a predominantly diffuse pattern in GL261 mouse brain tumors (33). Glioma TAMs originate from two independent sources: brain-resident microglia and BMDMs (30, 32). Transmembrane protein 119 (TMEM119) is exclusively expressed on cell-surface of microglia, and ionized calcium binding adaptor molecule 1 (Iba1) is a marker for macrophage. Our results showed that Iba1+ cells accumulated in the peritumoral area during tumor progression, while TMEM119^+^ microglia was located in the intratumoral area. Siglec-15^+^ TAMs was predominantly observed in the peritumoral area, indicating that Siglec-15^+^ TAMs might be originated from the bone marrow. Hence, we investigated whether Siglec-15 expression on BMDMs is upregulated by CM derived from several aggressive mouse tumor cell lines, including GL261, 4T1, MC38 and EG7. GL261-CM also increased Siglec-15 expression on peritoneal macrophages (PEMs), one type of tissue resident macrophage. However, once cells were activated by LPS and differentiated into M1-like macrophages, Siglec-15 expression was downregulated on both BMDMs and PEMs. After treatment with GL261-CM, addition to Siglec-15, proinflammatory M2 macrophage markers (ARG1 and CD206) were upregulated on macrophages. To further investigate the role of Siglec-15 in macrophage function, we generated Siglec-15 knockout macrophages. Consistent with a previous study, the expression of M2 phenotype macrophage biomarkers such as ARG1 and CD206 was significantly downregulated, after knocking out Siglec-15 (34). Interestingly, the costimulatory molecules CD86 and CD80 were upregulated, while the coinhibitory molecule PD-L1 was downregulated in Siglec-15-KO macrophage. As a result, the macrophage capabilities of migration, phagocytosis of apoptotic tumor cells, and antigen presentation to T cells were enhanced after Siglec-15 knockout. Cross-presentation by proinflammatory macrophages might be involved in local reactivation of effector and/or memory CD8^+^ T lymphocytes (35, 36). Our findings indicated that the impairment in CD8 T lymphocyte cross-priming by Siglec-15^+^ anti-inflammatory macrophages could be related to immune escape.

Increasing numbers of studies have demonstrated that anti-inflammatory macrophages are major contributors to therapeutic resistance in solid tumors (11, 37–39). In this study, after anti-PD-1 antibody therapy, GL261-bearing mice were classified into two groups based on therapeutic response (responder and nonresponder) defined by the median change in tumor size. Notably, the percentages of F4/80^+^CD206^+^ and F4/80^+^ Siglec-15^+^ macrophages in the TME were significantly higher in non-responders than in responders, whereas CD8 T-infiltration was decreased in the TME of non-responsive mice. Similar results were obtained in GL261-bearing mice treated with radiation therapy. More importantly, a negative correlation between PD-L1 and Siglec-15 expression was also found in the TME of GL261 model. In line with a previous study, S15^KO^ mice injected with GL261 tumor cells showed tumor growth inhibition compared to WT mice (13). Additionally, the proportion of tumor-free mice that achieved long-term survival was higher in S15 ^KO^ mice receiving an anti-PD-1 antibody than in anti-PD-1-treated S15^WT^ mice and untreated S15^KO^ mice, indicating the synergistic effect between anti-PD-1 and Siglec-15-targeted therapy. Collectively, our study results suggest that Siglec-15-mediated TAM immunosuppressive activity not only promotes tumor progression but also leads to therapeutic resistance in solid tumors. In a phase I trial of NC318, a Siglec-15 antibody, efficacy was not always seen in all patients, but one completely and the other partially response was encouraging in non-small cell lung cancer refractory to PD-1 blockade (25). However, the results from phase II trial of NC318 in several types of tumors was not satisfactory. Based on our findings, understanding the dynamic change in Siglec-15^+^ TAMs is critical to identify therapeutic timing of Siglec-15 antibody in combination with PD-1/PD-L1 blockade. Previous reports have reported that Siglec-15 is a rapidly internalized cell-surface antigen expressed on acute myeloid leukaemia blasts and bone-resorbing osteoclasts. Antibodies targeting Siglec-15 induced endocytosis and lysosomal degradation of cell surface Siglec-15 of myeloid leukaemia blasts and osteoclasts (40, 41). These results suggested that targeting TAMs with anti-siglec15 antibodies conjugated toxins might be a promising anti-tumor therapeutic strategy to decrease the population of TAMs or transform of TAMs into M1 proinflammatory macrophages.

## Conclusion

**Figure 7:**
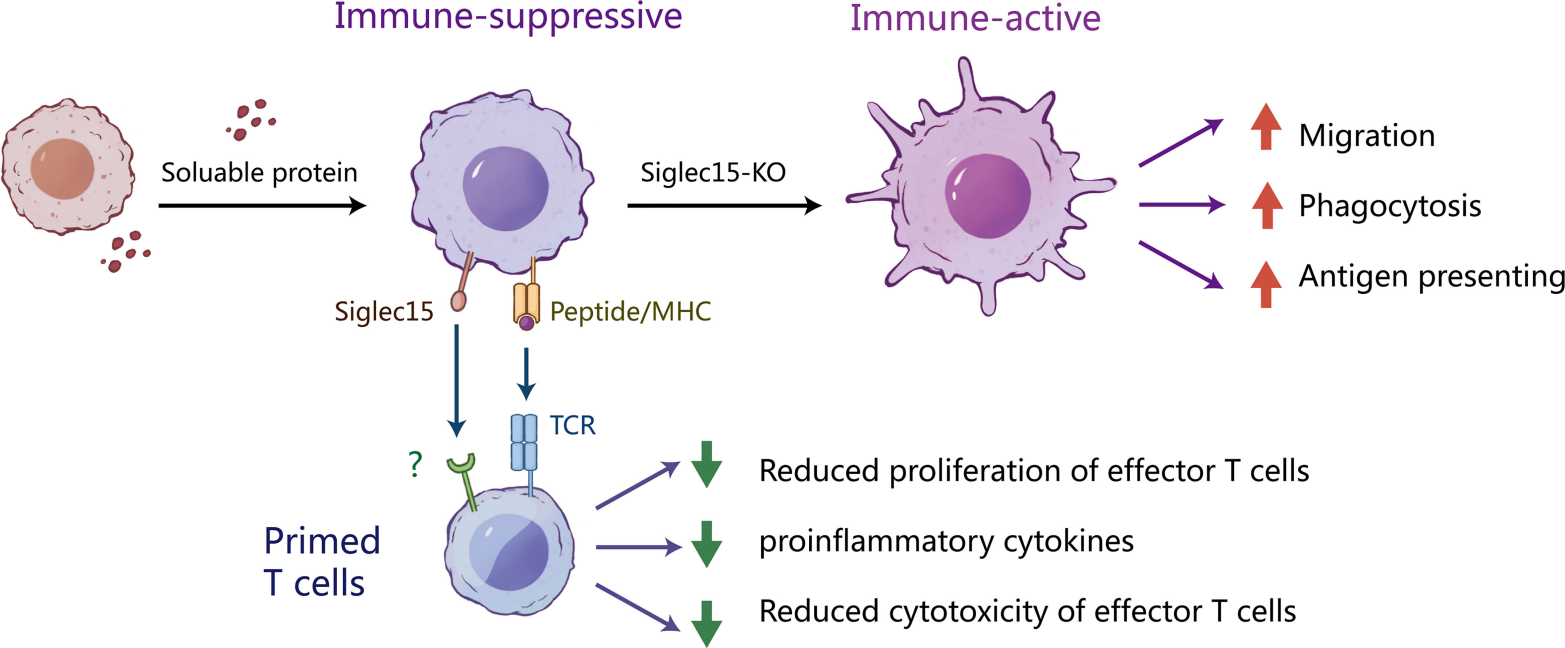
The potential mechanism by which Siglec-15 expressing tumor-associated macrophages suppress T cell function. We describe the mechanisms of the induction and suppressive function of Siglec-15 in the glioma TME. In the early stages of tumorigenesis, Siglect-15 expression on tumor associated macrophages (TAMs) is induced by soluble proteins released from tumor cells. As professional antigen presenting cells, macrophages act as scavengers of tumor-antigen. However, the existence of Siglec-15+ TAMs inhibits T-cell activation *via* recognition of antigenic peptides by TCR. Knockout of *Siglect15* promotes the macrophage migration, phagocytosis of apoptotic tumor cells and M1-like polarization, resulting in enhanced activation and proliferation of CD8 T cells. These findings provide new insights into the role of siglec-15 in tumor immune escape.

In summary, our study described a negative correlation between the Siglec-15 expression level and a poor prognosis in human glioma. Our study suggested that Siglec-15 was expressed predominantly on tumor-infiltrating macrophages, which are the major component promoting glioma progression and the development of therapy resistance. We further elucidated that Siglec-15 was involved in the inhibition of macrophage migration, phagocytosis and antigen presentation, resulting in impaired cross-priming of CD8 T cells (**Figure 7**). To our knowledge, the current study is the first to delineate a dynamic change in Siglec-15 positive macrophages in glioma TME, indicating that therapeutic timing is critical in immunotherapeutic targeting of Siglec-15 or other macrophage molecules.

## Materials and methods

### Acquisition of gene expression profiles from datasets

RNA-seq data for tumor tissues and corresponding clinical information in The Cancer Genome Atlas (TCGA) were downloaded through UCSC Xena (http://xena.ucsc.edu/). The expression of genes analyzed in normal tissues was collected from the Genome Tissue Expression (GTEx) database. The abbreviations and full names of TCGA cancers were listed in **Supplementary Table 1**. The expression levels of Siglec-15, CD68 and CD163 in different types of tumor and normal tissues were analyzed. Immune cell infiltration scores in glioma were evaluated using the Cell-type Identification By Estimating Relative Subsets Of RNA Transcripts (CIBERSORT) algorithm as described previously (42, 43). The gene expression correlation of SIGLEC15 with TGFB1 and MRC1 in glioma were estimated. Patients with glioma were divided into high and low groups according to the median gene expression level. The survival difference between high and low gene expression group was estimated by Kaplan Meier survival analysis.

### Patients

A total of 60 patients with glioma who underwent surgery at Fujian Medical University Union Hospital between April 2021 and April 2022 were included in this study. The clinical pathological features of the 60 patients with glioma are listed in **Supplementary Table 2**. None of these patients received preoperative radiotherapy or chemotherapy. Relevant clinical data were collected by retrospective review of the patients’ files, and follow-up data were available for all patients.

### Mice and cell lines

Female C57BL/6 mice (6–8 weeks old) were purchased from Beijing Vital River (Beijing, China). OT-I/CD45.2/Rag^-/-^ transgenic mice were bred in-house. *Siglec15*-knockout C57BL/6 mice (S15^KO^) were obtained from Gempharmatech Co. Ltd (Nanjing, China). Siglec-15-knockout mice and wild-type littermates (S15^WT^) were analyzed phenotypically.

4T1 mouse mammary tumor cells and EG7 mouse lymphoma cells were purchased from ATCC. GL261-luc murine glioma cells and MC38 mouse colorectal carcinoma cells were obtained from the laboratory of Dr. Lieping Chen (Yale University). Ana-1 mouse macrophages were obtained from the Kunming Institute of Zoology. Cells were cultured in RPMI-1640 or DMEM supplemented 10% FBS (Sigma Aldrich, USA).

### Mouse tumor models and treatment

C57BL/6 mice were anesthetized and injected with 1×10^5^ GL261-luc tumor cells in the right cerebral hemisphere of the brain using a standard stereotaxic instrument (RWD Life Science, USA). Established GL261-bearing S15^KO^ and S15^WT^ model mice were randomized into different groups, receiving craniocerebral radiation (4 Gy) on Day 7 using an RS-2000 Biological Irradiator (RadSource, Canada) or intraperitoneal injection of an anti-PD-1 mAb (clone G4, in-house) 200μg/mouse on Day 10. Tumor growth was monitored by bioluminescence imaging every 3-7 days using a Lumina XR Imaging System (PerkinElmer, USA). Tumor tissues were isolated on the indicated days for further analysis. The survival of the mice was monitored over a 28-day observation period.

### Immunohistological assays

Human glioma tissues were formalin-fixed and paraffin-embedded using standard procedures. For immunohistochemistry (IHC), sections were immunolabeled with primary antibodies, including a rabbit anti-Siglec-15 polyclonal antibody (#PA5-72765; Thermo Fisher, USA), a rabbit anti-PD-L1 monoclonal antibody (mAb) (#ab205921, Clone 28-8, Abcam, USA), anti-mouse CD4 (#ab271945, Clone EPR19514, Abcam, USA) and anti-mouse CD8 (#ab217344, Clone EPR21769, Abcam, USA), followed by processing with an anti-rabbit peroxidase kit (ImmPRESS™, USA) according to the manufacturer’s instructions. The intensity of positive staining in tissue sections was analyzed as the optical density (OD) using Image-Pro Plus 6.0 software (Media Cybernetics, USA), and the result was determined as the sum of 5 different fields (1 in the center and 4 in the periphery) with each section.

For immunofluorescence staining, sections were immunolabeled with an anti-Siglec-15, anti-CD3 (clone UCHT1, ThermoFisher, USA), anti-CD68 mAb (clone KPI, Thermo Fisher, USA), anti-Iba1 (#ab220815, Clone EPR16588, Abcam), or anti-TMEM119 (#ab234501, Clone 28-3, Abcam) as the primary antibody, followed by treatment with goat anti-mouse IgG-Alexa594 (#A-11005, Thermo Fisher, USA), goat anti-rat IgG-Alexa647 (#ab150155, Abcam, USA) or goat anti-rabbit IgG-Alexa488 (#A-11008, Thermo Fisher, USA), respectively. Finally, the slides were counterstained with DAPI to visualize cell nuclei (#D9542, Sigma Aldrich, USA). All slides were scanned using EVOS ™ FL auto imaging system (Thermo Fisher, USA), and the (OD) value of each fluorophore in the selected fields was analyzed by using Image-Pro Plus 6.0 software.

### Flow cytometry

Single-cell populations were isolated from fresh human glioma tissues and GL261 tumor tissues using a Gentle MACS mechanical dissociator in the presence of lysis buffers (Miltenyi Biotec, Germany). Tumor infiltrating immune cells were separated with Percoll (Sigma Aldrich, USA). For mouse cell analysis, cells were blocked with anti-mouse CD16/32 mAbs (TruStain fcX, USA) and subsequently stained with mAbs against mouse CD3e, CD4, CD8a, CD45, Siglc15, F4/80, CD206, PD-L1, CD25, CD69, MHC-I, MHC-II, CD80, CD86, TNF-α, IFN-γ, ARG1, iNOS and a death marker as well as matched isotype controls depending on the experiment. For intracellular staining, cells were fixed and permeabilized with Cytofix/Cytoperm buffer (BD Biosciences, USA) after surface marker staining. Single cells from human tumor tissues were blocked with human FcR blocking reagent (Miltenyi Biotec, USA) and then stained with antibodies against human Siglec-15, PD-L1, and death marker as well as isotype antibodies. These antibodies were obtained from Thermo Fisher or BD Biosciences. Stained samples were run on a BD FACSVerse™ and analyzed using FlowJo software (BD Biosciences, USA).

### Preparation of *Siglec15*-knockout Ana-1 cells

*Siglec-15* gene knockout Ana-1 cells (Ana-1-KO) were generated using a CRISPER/Cas9 system (Addgene, USA) *via* infection with a custom-made lentiviral vector containing the Cas9 gene and sgRNA targeting the sequence of mouse Siglec-15 (5′-caccggtgcccgcggaggtgaacg-3′). Infected cells were selected with puromycin (Sigma Aldrich, USA) for approximately 10 days before flow cytometry analysis, and then single cell clones were isolated by serial dilutions.

### Preparation of primary macrophages

To generate bone marrow-derived macrophages (BMDMs), bone marrow cells were isolated from the femurs of C57BL/6 mice and red blood cells were lysed with ammonium chloride potassium lysis buffer. Cells at a concentration of 1 × 10^6^ cells/ml were cultured in CM supplemented with 20 ng/ml recombinant mouse M-CSF (PeproTech, USA) for 7 days, and then the adherent cells (BMDMs) were harvested for further experiments. Primary peritoneal macrophages (PEMs) were isolated from the peritoneal cavity of mice.

### Macrophage proliferation assay

Ana-1-KO cells and Ana-1-WT cells were plated in 96-well plates at 1 × 10^5^ cells/well. The cell viability of cultured cells was quantified using Cell Counting Kit-8 (CCK-8) (Dojindo, Japan) and the SpectraMax^®^ i3x Multi-Mode Microplate Reader (Molecular Devices, USA).

### Macrophage migration assay

Ana-1-KO and Ana-1-WT cells were seeded in the upper chamber of an 8.0-μm transwell insert in a 24-well culture plate (Corning, USA) after culture under gradient serum starvation condition for 18 h. The lower chamber comprised RPMI 1640 medium contained10% FBS. After culture for 8 hours, the apical membrane of the upper chamber was fixed with methanol and stained with 0.1% crystal violet solution (Sigma Aldrich, USA) to evaluate cell migration. Five visual fields were randomly selected, and the OD value of each migrating cell was counted by Image-Pro Plus 6.0 software.

### Macrophage polarization assay

BMDMs isolated from the bone marrow of S15^KO^ or S15^WT^ mice were seeded in ultralow-attachment 24-well U-bottom plates (Corning, USA) at 1 × 10^5^ cells/well, and then cocultured with GL261 tumor cells at the ratio of 5:1 for 4 days. Macrophage surface markers were analyzed by flow cytometry.

### Macrophage phagocytosis assay

GL261 cells were stained with 3 µM 5,6-carboxyfluorescein diacetate succinimidyl ester (CFSE) (Sigma Aldrich, USA) and then subjected to 12 Gy of irradiation. The irradiated GL261 cells were incubated for 24 hours in complete medium before coculture with Ana-1 cells. Ana-1-KO cells and Ana-1–WT cells were stained with PE conjugated anti-mouse F4/80, followed by coculture with irradiated GL261 cells in a 96-well plate for 4 hours. The phagocytosis of CFSE labeled GL261 cells was analyzed by flow cytometry by quantifying the percentage of F4/80^+^CFSE^+^ Ana-1 cells.

### Real-time quantitative PCR (RT qPCR)

BMDMs were cultured with control CM or CM derived from several mouse tumor cells, including 4T1, GL-261, EG7 and MC38 cells. After incubation in CM for 24 hours. Ana-1-KO and Ana-1-WT cells were prepared as described above. All cells were collected for RNA extraction, cDNA synthesis and quantitative real-time PCR assays according to the manufacturer’s instructions (Takara, Japan). Primers are listed in **Supplementary Table 3**. *Siglec15* expression was standardized to the expression levels of the *Gapdh* gene using the 2^-△△Ct^ method. RT qPCR analyses were performed in triplicate.

### T cell proliferation and activation assay

CD8 T cells were magnetically isolated from the spleen of OT-I/CD45.2/Rag^-/-^ mice using the EasySep™ Mouse CD8 T-cell Enrichment Kit (STEMCELL Technologies, Canada) and labeled with 3 µM CFSE. Ana-1-KO and Ana-1-WT cells were loaded with 1 ng/ml OVA257-264 (SIINFEKL, H-2Kb) peptide at 37°C for 90 minutes and then washed 3 times with PBS. Then, 3 × 10^5^ OVA_257-264_-loaded Ana-1 cells were cocultured with CFSE-labeled T cells at a ratio of 1:5 for 3 days, and then T-cell proliferation and activation was assessed by evaluating CFSE dilution and the expression of activation markers (CD25, CD69, TNF-α and IFN-γ) using flow cytometry.

### Statistical analysis

GraphPad Prism version 9.0 was used to generate plots and perform additional statistical analyses. All data are presented as the mean ± SEM or mean ± SD of a single experiment, representative of two or three independent experiments. A two-tailed unpaired t test or two-way ANOVA was used to determine the statistical significance of intergroup difference. Correlation analyses were performed using Pearson coefficient analysis. Statistically significant results were defined as follows: ns, no significance; ### *P*<0.001; *P < 0.05; **P < 0.01; ***P < 0.001 and ****P < 0.0001.

## Data availability statement

The original contributions presented in the study are publicly available. This data can be found here: https://doi.org/10.6084/m9.figshare.22011368.

## Ethics statement

The studies involving human participants were reviewed and approved by the Research Ethics Committee of Fujian Medical University Union Hospital (No. 2021KY044). The patients/participants provided their written informed consent to participate in this study. The animal study was reviewed and approved by Fujian Medical University Institutional Animal Care and Use Committee (IACUC, No. 2019-138).

## Author contributions

QC and QZ conceived and designed the study; QC and BC performed the experiments; QC and CW cooperated in the establishment of tumor models; LH, QW, and YZ provided technical guidance; QC and QZ analyzed the data and drafted the manuscript; QZ supervised the study and reviewed the manuscript. All authors contributed to the article and approved the submitted version.
