# Supplementary material for: Dynamic change in Siglec-15 expression in peritumoral macrophages confers an immunosuppressive microenvironment and poor outcome in glioma
Source: Front Immunol. 2023 May 10;14:1159085. doi: 10.3389/fimmu.2023.1159085 (PMC10206144; doi:10.3389/fimmu.2023.1159085)
Supplement: Supplementary file 1 [file Presentation_1.pdf]

Supplementary Material

Dynamic change of Siglec-15 expression in peritumoral macrophages confers an immunosuppressive microenvironment and poor outcome in glioma

Quan Chen[1, 2†], Bing-kun Chen [1, 3†], Chun-hua Wang[1†], Li Hu[1], Qiong-wen Wu[1], Yanyang Zhu[1], Qiu-Yu Zhang[1, 3*]

*** Correspondence:** Qiuyu Zhang, Institute of Immunotherapy, Fujian Medical University, Fuzhou, Fujian, 350102, China; phone: +86-15080008791; email: qiuyu.zhang@fjmu.edu.cn

[†] These authors contributed equally to this work and share first authorship

1 Supplementary Figures and Tables

1.1 Supplementary Figures

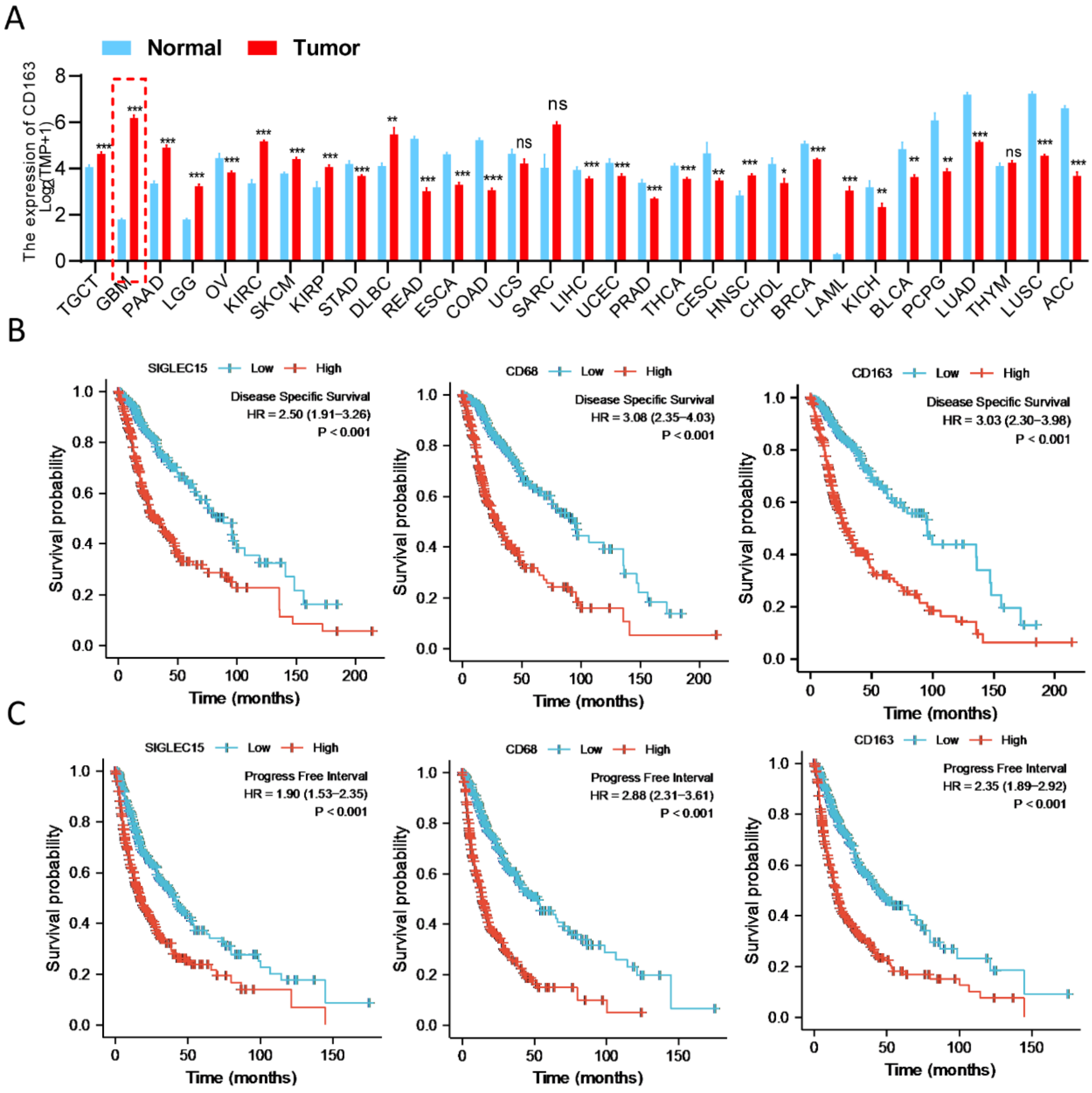

Supplementary Figure 1. The relationships between Siglec-15 expression and survival time of patients with glioma. (**A**). The meta-analysis of CD 163 expression levels in 31 types of human cancers and their corresponding normal tissues based on TCGA and GTEx databases. (**B-C**). The association between Siglec-15 mRNA expression levels and Disease specific survival (B) or Progress free survival of glioma patients (n = 695). SIGLEC15 expression levels were binned into high and low based on median cutoff, and survival curves were compared using log-rank (Mantel–Cox) test. The data represent the mean ± SEM and analyzed by two-tailed unpaired t-test. ns, no significance; * $P < 0.05$, ** $P < 0.01$, *** $P < 0.001$.

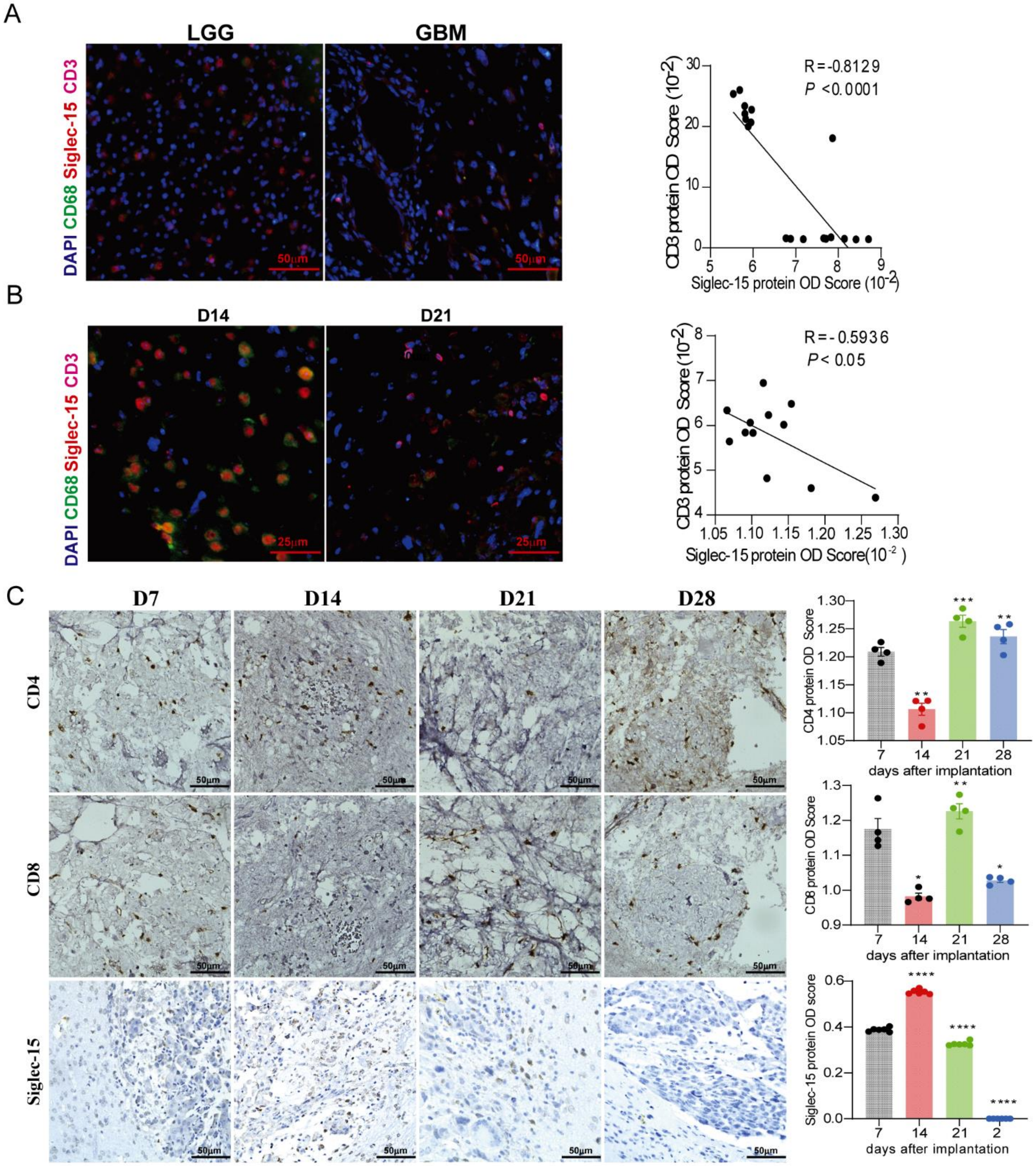

Supplementary Figure 2. The association of Siglec-15 expression and T cell infiltration in tumor tissues. (A-B). Representative images and statistical data of immunofluorescent staining of CD3 and Siglec-15 in tumor tissues of glioma and GL261 allografts. (**C).** Representative images and statistical data of immunohistochemical staining of CD4, CD8 and Siglec-15 in GL261 allografts tissue on day7, day14, day21 and day28 after tumor implantation. Scale bar: 50 μm, ×200 magnification; 25 μm, × 400 magnification. The expression score of CD3, CD4, CD8 and Siglec-15 protein in tumor tissues was calculated using OD score quantified by ImageJ software (NIH). Data are representative of three independent experiments. The data represent the mean ± SD and analyzed by two-tailed unpaired t-

test. LGG: lower grade glioma; GBM: glioblastoma. * $P < 0.05$, ** $P < 0.01$, *** $P < 0.001$, **** $P < 0.0001$.

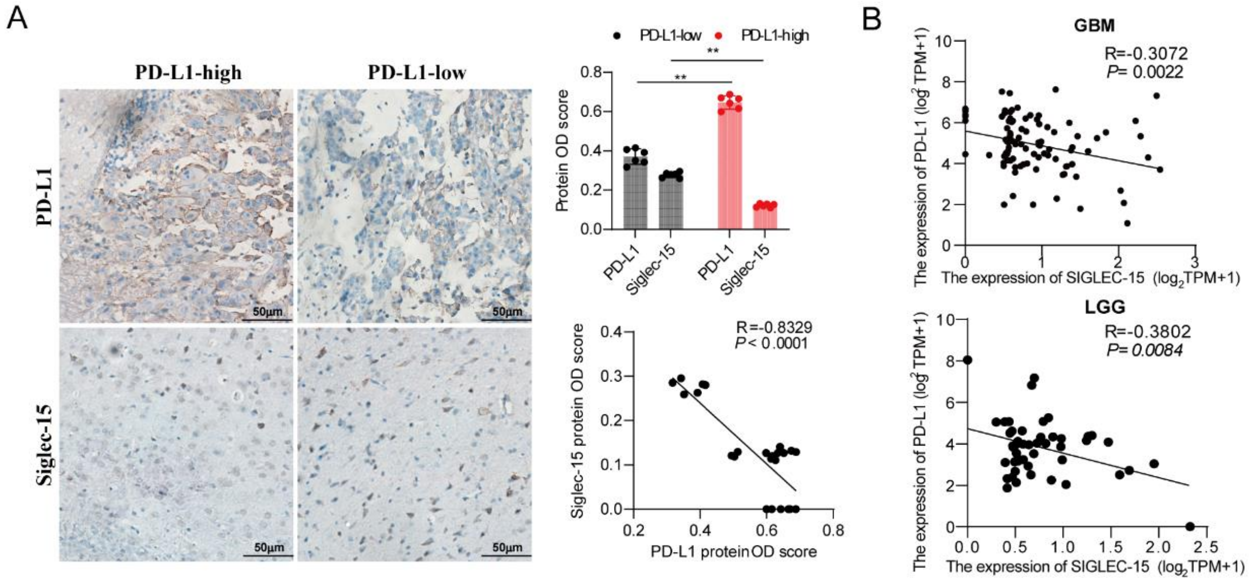

Supplementary Figure 3. Siglec-15 expression was negatively associated with PD-L1 expression in tumor tissues of giloma. (A). Representative images of immunohistochemical staining of Siglec-15 and PD-L1 in GL261 allograft tissues. Scale bars, 50 μm. Siglec-15 expression was compared between high and low groups characterized by median OD score of PD-L1 expression. The correlation between OD score of Siglec-15 and PD-L1. Data are mean ± SD from two independent experiments. P values by a two-tailed unpaired t-test, ** $P < 0.01$. (**B).** The correlation of mRNA expression levels between Siglec-15 and PD-L1 in recurrent glioblastoma (upper graph, n = 97) and recurrent low-grade glioma (lower graph, n = 47) by meta-analysis of TCGA databases. LGG: lower grade glioma; GBM: glioblastoma. ** $P < 0.01$.

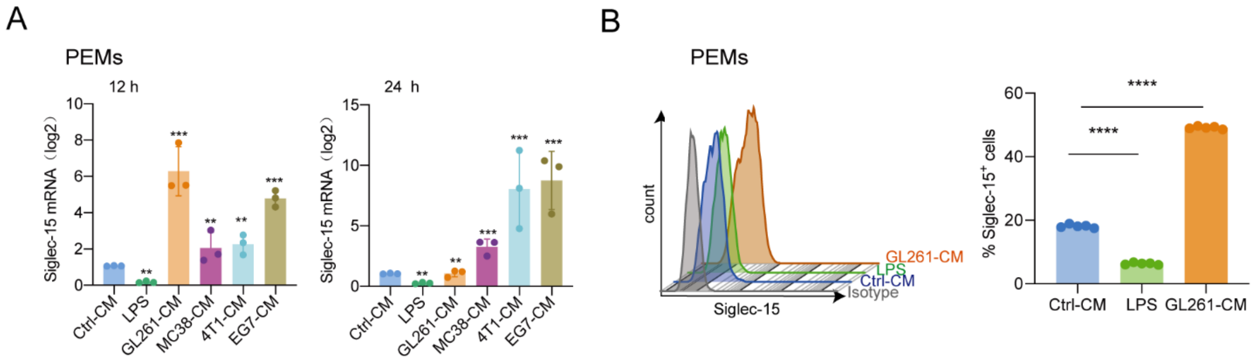

Supplementary Figure 4. Siglec-15 expression was induced in peritoneal macrophage. (A). The expression of Siglec-15 in PEMs was induced by cell culture media of various types of tumor cell lines. PEMs was incubated with 1ug/ml LPS or the media of 4T1, GL261, MC38 and EG-7 cells or control medium for 12 h and 24 h, Siglec-15 mRNA levels was determined by RT-PCR. **(B).** Siglec-15 expression on PEMs was analyzed by flow cytometry in the presence of LPS or cell media of GL261. PEMs: Peritoneal macrophages. LPS: Lipopolysaccharides; CM: Cell culture media. The data represent the mean ± SD and analyzed by two-tailed unpaired t-test. ** $P < 0.01$, *** $P < 0.001$, **** $P < 0.0001$.

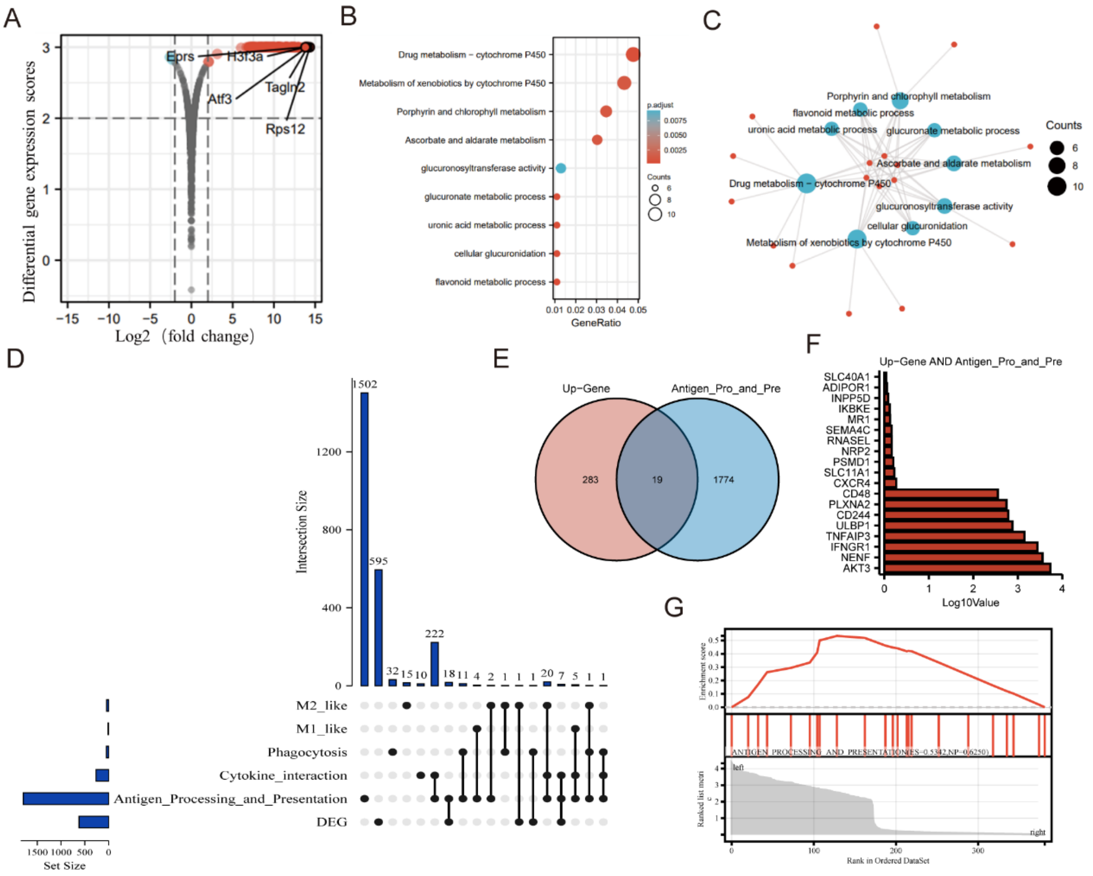

Supplementary Figure 5. Differentially expressed genes of Siglec15 knockout and control Ana-1 cells. The identification of gene groups and enriched pathways based on differentially expressed genes in Siglec15 knockout and control Ana-1 cells (S15KO Ana-1 and WT Ana-1) was performed by the GSEA analysis. (**A**) Volcano plot of gene expression of S15KO Ana-1 and WT Ana-1 cells. The red and green dots represent up- and downregulated DEGs (differentially expressed genes), respectively. (**B**). The differentially expressed genes of enriched biological functions between S15KO Ana-1 and WT Ana-1 were analyzed by Gene Ontology (GO). The number of enriched DEGs in the pathway is indicated by the circle area, and the circle color represents the ranges of the p-adjust. (**C**). STRING interaction network analysis of the pathways. Each filled node denotes a pathway; edges between nodes indicate interactions between pathways of the corresponding genes. (**D**). UpSet plot of intersections between sets of differentially expressed genes across M1-like, M2-like, phagocytosis, cytokine interaction, Dig and antigen processing and presentation. The upper bar chart indicates the intersection size between sets of genes within each intersection. Set size indicates the total number of significant genes in the S15KO Ana-1 versus WT Ana-1 cells. Dark connected dots on the bottom panel indicate which substrates are considered for each intersection. (**E**). A Venn diagram showing the overlap between differentially expressed genes and antigen-presentation-related genes. (**F**). 19 upregulated genes associated with antigen processing and presentation in Ana-1-KO compared with Ana-1-WT. (**G**). Gene Set Enrichment Analysis (GSEA) plot in Ana-1-KO versus Ana-1-WT cells. Antigen processing and presentationignatures was strongly correlated with target genes. p-values were obtained by two-tailed unpaired t-test or Pearson correlation analysis. The primary data can be found here: https://doi.org/ 10.6084/m9.figshare.22011368.

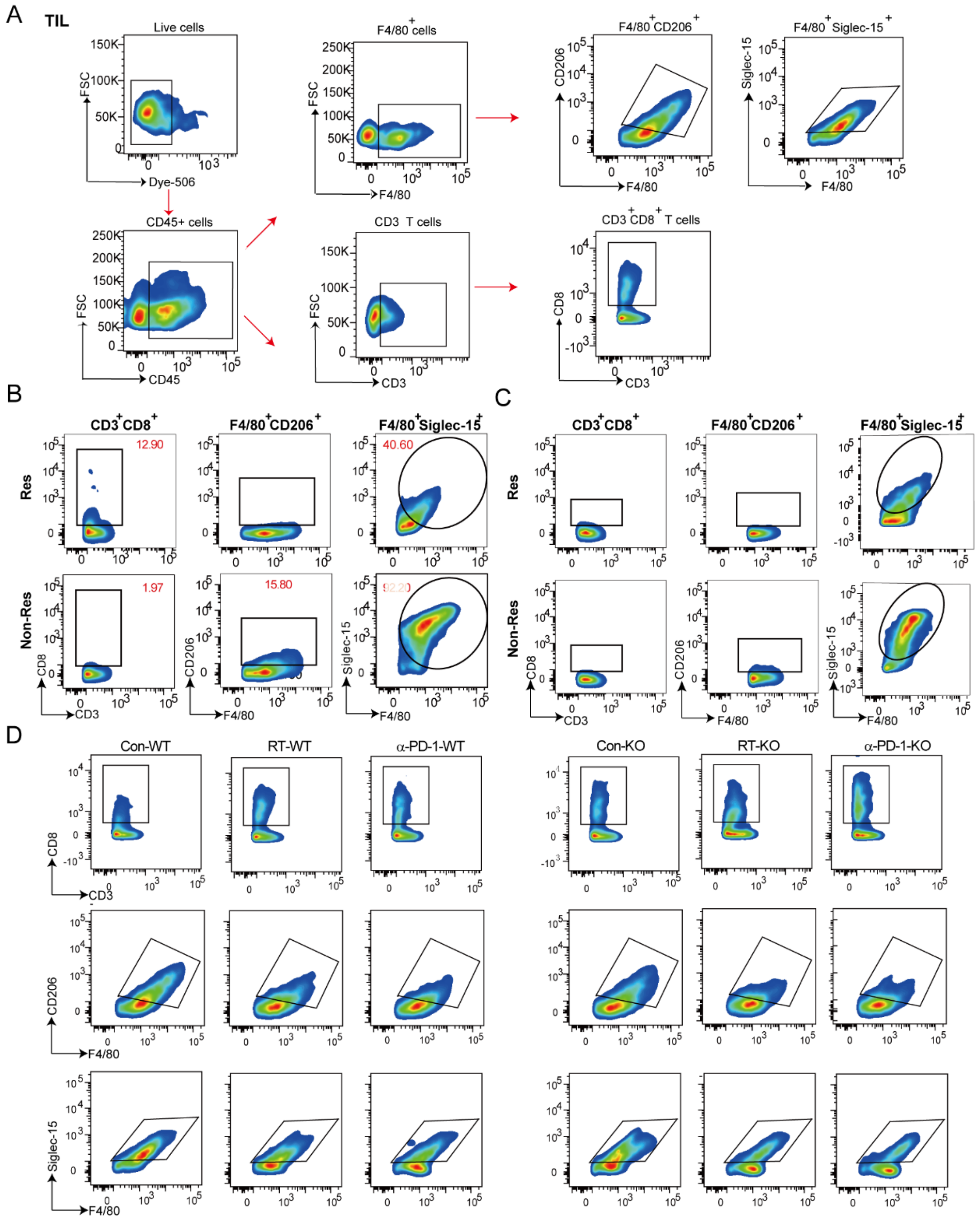

Supplementary Figure 6. The percentage of tumor-infiltrating immune cells in different groups of radiation therapy or anti-PD-1 therapy. (**A).** Gating strategy for flow-cytometry analysis of the live cells, CD45^{+} cells, living cells, T cells, CD3^{+}CD8^{+} T cells, TAMs and F4/80+Siglec-15^{+} cells. **(B-D)**. Representative plots of percentages of CD3^{+}CD8^{+} T cells, F4/80^{+}Siglec-15^{+} cells and F4/80^{+}CD206^{+} were analyzed by flow cytometry in tumor tissues of different groups. Res: response; Non-Res: non-response; WT-Con: wide type mice with no treatment; WT-RT: wide type mice treated with radiation; KO-RT: knockout mice treated with radiation; WT-α-PD-1: wide type mice treated with anti-PD-1 antibody; KO-α-PD-1: knockout mice treated with anti-PD-1 antibody.

1.2 Supplementary Tables

Supplementary Table 1. Abbreviations and full names of cancers in TCGA

Abbreviations	Full names
ACC	Adrenocortical carcinoma
BLCA	Bladder Urothelial Carcinoma
BRCA	Breast invasive carcinoma
CESC	Cervical squamous cell carcinoma and endocervical adenocarcinoma
CHOL	Cholangio carcinoma
COAD	Colon adenocarcinoma
DLBC	Lymphoid Neoplasm Diffuse Large B-cell Lymphoma
ESCA	Esophageal carcinoma
GBM	Glioblastoma multiforme
HNSC	Head and Neck squamous cell carcinoma
KICH	Kidney Chromop hobe
KIRP	Kidney renal papillary cell carcinoma
LAML	Acute Myeloid Leukemia
LGG	Lower Grade Glioma
LIHC	Liver hepatocellular carcinoma
LUAD	Lung adenocarcinoma
LUSC	Lung squamous cell carcinoma
MESO	Mesot helioma
OV	Ovarian serous cystadenocarcinoma
PAAD	Pancreatic adenocarcinoma
PCPG	Pheochromocytoma and Paraganglioma
PRAD	Prostate adenocarcinoma
READ	Rectum adenocarcinoma
SARC	Sarcoma
SKCM	Skin Cutaneous Melanoma
STAD	Stomach adenocarcinoma
TGCT	Testicular Germ Cell Tumors
THCA	Thyroid carcinoma
THYM	Thymoma
UCEC	Uterine Corpus Endometrial Carcinoma
UCS	Uterine Carcinosarcoma
UVM	Uveal Melanoma

Supplementary Table 2. Patients' characteristics

Clinico-pathological Factors (N=60)	Patient number	Percentage (%)
Age		
≤50	31	51.7
>50	29	48.3
Gender		
male	32	53.3
female	28	46.7
WHO grade		
G1	7	11.7
G2	17	28.3
G3	16	26.7
G4	20	33.3
Ki67 expression (positive ratio)		
≤5%	18	30
5%-10%	27	45
>10%	15	25
Tumor size（cm）		
≤2	10	16.7
2-5	34	56.7
>5	16	26.6

Supplementary Table 3. Mouse gene primers for realtime PCR

Gene	Forward	Reverse
gapdh	5′-aagcccatcaccatcttcca-3′	5′-cctgcctcaccaccttcttg-3′
siglec-15	5′-cagcaccgagatgttgacga-3′	5′-acgatcgctatgagagtcgc-3′
ccl2	5′-gctacaagaggatcaccagcag-3′	5′-gtctggacccattccttcttgg-3′
ccl5	5′-actgcatctgccctaaggtctt-3′	5′-tgcttgaggtggttgtggaa-3′
cxcl9	5′-gtccgctgttcttttcctcttg-3′	5′-ggtgctgatgcaggagcat-3′
cxcl10	5′-gtccgctgttcttttcctcttg-3′	5′-ggtgctgatgcaggagcat-3′
cxcl11	5′-gaccaggttgggcaaagaga-3′	5′-ggcatcctggacccacttct-3′
